# Bank Specific Risks and Financial Stability Nexus: Evidence From Pakistan

**DOI:** 10.3389/fpsyg.2022.909141

**Published:** 2022-06-24

**Authors:** Zhengmeng Chai, Muhammad Nauman Sadiq, Najabat Ali, Muhammad Malik, Syed Ali Raza Hamid

**Affiliations:** ^1^Faculty of Management and Economics, Kunming University of Science and Technology, Kunming, China; ^2^Faculty of Management Sciences, Hamdard University, Islamabad, Pakistan; ^3^School of Finance and Economics, Jiangsu University, Zhenjiang, China

**Keywords:** financial stability, bank risk, credit risk, funding risk, bank size, liquidity risk

## Abstract

This article investigates the nexus between bank-specific risks and the financial stability of the banks for a panel data set of 15 scheduled banks in Pakistan over a 12-year period from 2009 to 2020. Using the fixed-effect model, the study result shows that bank-specific risks, i.e., credit risk and liquidity risk are detrimental to bank stability, whereas funding risk has no significant impact on bank stability. Besides these, bank size has also a negative impact on bank stability, whereas the return on assets (ROA) revealed a positive influence. To ensure stability, bank management should establish policies that confirm secure loan granting and timely reimbursement from customers to minimize the credit risk. Besides this, management should keenly observe the liquidity position and should also effectively mobilize the customer deposits to attain financial stability.

## Introduction

The global financial crisis of 2007/2008 directed the attention of investors, policymakers, and governments toward the financial stability of the financial sector (Viñals et al., 2013; Shukla, 2014; Adusei, 2015; Kim et al., 2020). The need for this proposition was felt due to the interconnection of the financial sector and the economic development of any country. Several studies observe this interconnection and find that the financial sector not only mobilizes the financial resources for the economy but its stability is significant for the economic development of the country (King and Levine, 1993; Athanasoglou et al., 2008; Shukla, 2014). Husain (2005) stated that “no economy can grow and improve the living standards of its population in the absence of a well-functioning and efficient financial sector.” Several countries achieve their economic growth with the support of their well-established banking system. However, China appeared like an economic superpower with the support of its banking system (Gupta and Kashiramka, 2020).

Since the global financial crisis of 2007/2008 and the recent Covid-19 pandemic, numerous studies have been conducted to disclose the different factors explaining the financial stability of the banking sector (Adusei, 2015; Diallo et al., 2015; Ghenimi et al., 2017; Ali et al., 2019; Barra and Zotti, 2019; Zaghdoudi, 2019; Djebali and Zaghdoudi, 2020; Gupta and Kashiramka, 2020; Kim et al., 2020; Mutarindwa et al., 2020; Nair and Anand, 2020; Feghali et al., 2021; Lesmana, 2021; Malik et al., 2021). These studies used several factors as determinants of banks stability, such as funding risk (Adusei, 2015), credit, liquidity, and operational risk (Diallo et al., 2015; Zaghdoudi, 2019), Corporate governance (Subhani and Zeb, 2021), Governance quality and financial inclusion (Malik et al., 2021), Bank size (Ali et al., 2019) and Accounting (Bischof et al., 2020). Besides these factors, several studies used macroeconomic factors as determinants of bank stability, such as political stability (Djebali and Zaghdoudi, 2020), bank market structure (Feghali et al., 2021), monetary policy (Nair and Anand, 2020), corruption (Ali et al., 2019), and economic policy uncertainty (Phan et al., 2020). These studies are significant for bank management to comprehend the role of these factors in the financial stability of banks. Among these factors, risk management is the primary concern of policymakers because of its major role in bank stability (Lassoued et al., 2016). Most of the studies examine credit, liquidity, and operations risks as major risks for financial stability (Djebali and Zaghdoudi, 2020). These studies disclosed divergent results, which kept the debate on the relationship between risk factors and financial stability inconclusive. This study is an attempt to disclose the relationship between risk factors and financial stability from the perspective of Pakistan.

Pakistan is a developing country and its financial sector is comprised of 95% of banks (Husain, 2005). In developing countries, banks play a key role in funding and stimulating economic growth (Djebali and Zaghdoudi, 2020). Hence, the improved condition of the banking sector influences the growth of the country (Husain, 2005). The present study is an attempt to find the factors explaining the financial stability of the banking sector in Pakistan. To accomplish it, we use bank-specific risks as a determinant of the financial stability of the banks. Bank-specific risks include operational, credit, and liquidity risks (Hunjra et al., 2020), and financial institutions mostly face these types of risks (Hussain and Al-Ajmi, 2012). Hence, it is significant to investigate these types of risks in relation to a bank's financial stability. The present study is using credit risk, liquidity risk, funding risk, and interaction of credit and liquidity risks as bank-specific risks in relation to the financial stability of the banks. The present study only used the conventional banks with no missing data for the selected period. However, a previous study mixed both Islamic and conventional banks (Ali and Puah, 2018). This study is contributing to the literature by investigating the impact of bank-specific risks on the financial stability of the conventional banking system of Pakistan.

This article is arranged as follows. The first section comprises a brief introduction of the study, and a relevant literature review is present in section two. Section three outlines the variables and their specification and also discusses the econometric model and estimation procedure. Section four reports the study results and in the last section, we presented the conclusion, policy implication, and recommendations for future research.

## Literature Review

Financial stability refers to the capability of a financial organization to accelerate its economic procedures, absorb shocks, and control risk (Schinasi, 2004). After the global financial catastrophe of 2007/2008, many studies examined the factors explaining the financial stability of banks Babar et al., 2019; Kim et al., 2020). These researchers examine many internal factors, such as “liquidity risk,” “credit risk,” and “operational risk” (Ghenimi et al., 2017; Amara and Mabrouki, 2019; Khemais, 2019; Djebali and Zaghdoudi, 2020). The following are some studies that have attempted to elucidate the financial stability of banks.

## Liquidity Risk and Financial Stability

Liquidity is referred to as the short-term paying capacity of firms. Commercial banks are obligated to hold a sufficient amount of liquid assets that can readily be changed to cash. While company's inability to meet its short-term obligations instigated liquidity risk. A study revealed that during the financial disaster of 2008, banks with liquidity risk faced financial instability and influenced other banks, as well as the economy (Mishra et al., 2012; Shukla, 2014). Gupta and Kashiramka (2020) directed a study to understand the influence of liquidity creation on the financial stability of commercial banks in India. This study revealed that “liquidity creation strengthens the stability of the banks.” Besides these, many studies find a significant negative influence of liquidity risk on banks' financial stability (Imbierowicz and Rauch, 2014; Mensi and Labidi, 2015; Hakimi et al., 2017; Khemais, 2019). During the literature review, we find some contradictory results, such as Zaghdoudi (2019) who finds a significant positive influence of liquidity risk on the stability of Tunisian banks. Besides these results, some other studies also found similar results (Ali and Puah, 2018). However, some studies find no influence of liquidity risk on financial stability (Amara and Mabrouki, 2019).

Based on the above discussion, we hypothesized:

***H1***. *Liquidity risk has a significant impact on financial stability*.

## Credit Risk and Financial Stability

Credit risk is referred to the possibility of failure of a bank borrower to pay its dues as per agreement. If a borrower defaults on his or her obligations, the credit risk level for financial institutions rises, which results in lower profits (Tan and Floros, 2012). Numerous studies have been directed to explore the influence of credit risk on bank stability and find diverse results. Among these, many studies revealed that credit risk reduces the banks' stability (Acharya and Mora, 2013; Rajhi and Hassairi, 2013; Khemais, 2019; Djebali and Zaghdoudi, 2020). Besides these, many studies find no significant influence of credit risk on banks' stability (Amara and Mabrouki, 2019; Zaghdoudi, 2019). Based on the above discussion, we hypothesized:

***H2***. *Credit risk has a significant impact on financial stability*.

## Funding Risk and Financial Stability

Adusei (2015), defined funding risk as “the probability that the deposit mobilization strategies of a rural bank will fail or the probability that depositors of a rural bank will withdraw their deposits, resulting in the deterioration of the bank's deposits which compels it to fall on equity sources of funding.” The funding risk variable was initially used by Adusei (2015) in relation to financial stability and revealed that it positively influences the financial stability of banks. Further, he revealed that if a bank demonstrates a long-term strategy of deposit utilization, it has a better chance of being steady than its rivals. In the literature, we find very limited studies using funding risk as a determinant of financial stability. However, whatever studies we find show a significant positive impact of funding risk on financial stability (Ali et al., 2019).

Based on the above discussion, we hypothesized:

***H3***. *Funding risk has a significant impact on financial stability*.

## Data and Methodology

### Sample Specification

Our sample comprises 15 Scheduled banks in Pakistan with a 12-year observation period from 2009 to 2020. In the present study, we only used conventional banks and among these excluded those banks that had some missing observations for the selected period.

### Variables Specification

#### Dependent Variable

##### Financial Stability

Financial stability is used as a dependent variable for the current study and is measured using a widely used proxy, i.e., the *z*-score. It measures the distance to insolvency (Rojas-Suarez, 2002) and is measured as follows:


$$\begin{array}{l} Z-Score=\frac{ROA+ETA}{\sigma \left(ROA\right)}, \end{array}$$

where ROA represents the profitability ratio, i.e., “Return on Assets,” ETA denotes the “level of capitalization” of the bank i.e., “Equity to Total Assets,” and σ *ROA* is the standard deviation of the *ROA*. A higher value of the *z*-score represents a lesser chance of insolvency risk and more bank stability (Uhde and Heimeshoff, 2009; Jeon and Lim, 2013; Li et al., 2017).

#### Independent Variables

The present study used different bank-specific risk factors as independent variables. The explanation of these variables is given in Table 1. Credit Risk (CR) is measured as a “Non-performing loan to total Advances.” It represents the chance of failure of a bank borrower to pay its dues as per agreement. A greater value of this ratio represents a greater level of credit risk (Natsir et al., 2019). Liquidity risk is measured as “Liquid Assets/Total assets.” It referred to the short-term paying capacity of firms. The higher value of this ratio represents a higher level of liquidity risk.

**Table 1:** Description of variables.

**Symbol**	**Variables**	**Measures**
BSTAB	Bank Stability	$Z-Score=\frac{ROA+ETA}{\sigma \left(ROA\right)}$
CR	“Credit Risk”	“Non-performing loan to total Advances”
LR	“Liquidity risk”	“Liquid Assets /Total assets”
FRISK	Funding risk	$Z-\text{score }\left(\text{FUNDRISK}\right)=\;\frac{DEP/TA_{i,t}+E/\;TA_{i,t}}{\sigma \left(DEP/TA_{i,t}\right)}$
SIZE	“Bank size”	“ln(total assets)”
“ROA”	“Return on Assets”	$\frac{\text{Net Profit after tax}}{Total\;assets}\times \;100$

Besides these variables, the present study also used funding risk as to the independent variable. Adusei (2015) used *Z*-score to measure the funding risk and explained that it measures “the number of deviations customer deposits would have to fall to compel the bank to wipe out equity finance.” The higher value of funding risk (*z*-score) represents “increasing efforts in mobilizing customer deposit” which results in funding stability (Ali and Puah, 2018). It is significant to study funding risk because retail banks rely on client deposits to sustain their operations (Köhler, 2015).

It is computed as follows;


$$\begin{array}{l} Z-\text{score }\left(\text{FUNDRISK}\right)=\;\frac{DEP/TA_{i,t}+E/\;TA_{i,t}}{\sigma \left(DEP/TA_{i,t}\right)} \end{array}$$

In the above equation “*DEP*/*TA*” is representing “Deposit to total assets” *E*/*TA* denotes the “level of capitalization” of the bank i.e., “Equity to Total Assets” and these are further divided by the standard deviation of “Deposit to total assets.”

#### Control Variables

The present study used bank size and ROA as control variables. These variables are also used as control variables in many studies investigating the association of liquidity and credit risk with bank profitability and stability (Munteanu, 2012; Tan, 2015; Ghenimi et al., 2017).

## Modeling Financial Stability (Z-score)

To examine the results, we use the following equation:


$$\begin{array}{l} Z-Score_{it}=\;\beta_{{}^{\circ}}+\beta_{1}CR_{it}+\beta_{2}\;LR_{it}+\beta_{3}FRISK_{it}+\beta_{4}CR_{it}\\ \;*\;LR_{it}+\beta_{5}Size_{it}+\;\beta_{6}ROA_{it}+\;\varepsilon_{it} \end{array}$$

In the above equations *LR*_*it*_, *CR*_*it*_ represents liquidity and credit risk respectively. Where *FRISK* denotes the “funding risk” ROA “Return on assets” where $CR_{it}^{*}LR_{it}$ represents the interaction of liquidity and credit risk.

### Estimation Procedure

To investigate the nexus between bank-specific risks and the financial stability of the banks in Pakistan, the present study used the Hausman test to select a suitable model for our study. Based on the Hausman test result, we used the fixed-effect model for our study. Before conducting the Hausman test, the study also conducted some diagnostic tests, such as multicollinearity analysis and heteroscedasticity test. To check the problem of multicollinearity, the study used correlation analysis and variance inflation factor (VIF). Whereas to check the problem of heteroscedasticity study used the white test.

In Table 2, the descriptive statistics of the data are shown. The study used 180 observations from 15 banks in Pakistan. The mean value of the *z*-score (which represents the banks' stability) is very low as compared to values reported in the literature from different countries, like Adusei (2015) reported 2.29 the value of the *z*-score of Ghana banks while (Zaghdoudi, 2019) reported 20.87 for Tunisian banks, which is very high and shows the stability of banks. Our value of 0.9161 revealed that our banks are less stable. Besides this, the mean value of ROA is also very low, which shows less productivity of assets. Along with this, the mean value of funding risk, credit risk, and liquidity risk is also very high, which reveals the high internal risk of commercial banks in Pakistan.

**Table 2:** Descriptive statistics.

**Variable**	**“Mean”**	**“Std. Dev”**	**“Min”**	**“Max”**
Z-Score (Stability)	0.916	1.87	−9.30477	9.42
CR	10.58	7.26	0	51.56
LR	8.50	2.41	4.69	15.8
ROA	0.943	0.99	−5.41	3.06
Funding risk	75.63	6.58	59.45	90.86
Size	19.93	1.24	16.14	22.02
CRLR	−0.049	0.75	−3.91	2.08

Table 3 shows the VIF value and correlation coefficient results. VIF values are used to check for the possible problem of multicollinearity. As the VIF values are <10, this shows multicollinearity is not a problem in the model. The correlation coefficient shows the potential relationship between variables. The result divulged that only ROA has a significant positive association with Stability. Aside from that, CR, FRISK, and LR have a negative correlation coefficient with stability, however, not significant at a 5% level of significance. These results show the weak form of association between risks and financial stability of commercial banks.

**Table 3:** The variance inflation factor (VIF) and correlation coefficient.

**Construct**	**VIF**	**1/VIF**	**Stability**	**CR**	**LR**	**ROA**	**SIZE**	**FR**	**CRxLR**
Stability	1.44	0.6944	1.0000						
CR	1.49	0.6723	−0.0956	1.0000					
LR	1.24	0.8039	−0.0866	0.0003	1.0000				
ROA	1.33	0.7522	0.5640*	−0.1971*	0.0675	1.0000			
SIZE	1.40	0.7153	0.1076	−0.2066*	0.2193*	0.4742*	1.0000		
FR	1.19	0.8382	−0.0275	0.2350*	0.3083*	−0.0088	0.0724	1.0000	
CRxLR	1.48	0.6771	0.1076	−0.5421*	−0.0357	0.2466*	0.2190*	−0.1812*	1.0000

“*Significant at the 0.05 level”*.

Table 4 reports the result of white test, as it is conducted to check the possible problem of heteroscedasticity. As the significance value is >0.05, it shows that heteroscedasticity is not a problem in the model (White et al., 1980).

**Table 4:** White test for heteroscedasticity.

	**chi2**	**df**	***p***
Heteroscedasticity	25.97	27	0.5205

Table 5 reports the result of the fixed-effect model. We used the Hausman test to select a suitable model for our study. Based on the Hausman test result, we used a fixed-effect model for our study. The result shows that bank-specific risks i.e., credit risk and liquidity risk have a negative impact on bank stability, whereas funding risk has no significant impact on bank stability. Besides these, bank size has also a negative impact on bank stability, whereas ROA revealed a positive influence. These findings are consistent with the literature (Imbierowicz and Rauch, 2014; Adusei, 2015; Ghenimi et al., 2017). Acharya and Mora (2013) revealed that banks that failed during the global financial crisis of 2007/2008 suffered from liquidity problems. He further elaborated that both credit and liquidity risk can push the bank to default. Rajhi and Hassairi (2013) also revealed that if banks are not ensuring the secure loan granting and timely reimbursement from customers it reduces the stability of the banks. Adusei (2015) described that “bank credit risk should have a negative impact on bank stability due to poor standards in lending rates” Furthermore, he reported the negative consequence of liquidity risk on bank stability.

**Table 5:** Fixed effect model.

**Variables**	**Coefficient**	***t*-statistics**	***P*-value**
CR	−0.0573677	−2.04	0.043
LR	−0.1974637	−2.59	0.010
FR	0.0189639	0.85	0.398
CR*LR	−0.2176038	−1.03	0.305
ROA	1.19059	7.48	0.000
Size	−0.9837046	−3.53	0.001
_cons	20.23684	3.24	0.001
Dependent variable: Financial Stability			

*F-stats = 13.01; Prob > F = 0.0000; Hausman test: X^2^= 41.43; Prob = 0.0000*.

Furthermore, our study finds that funding risk has no significant influence on their stability. These findings are not consistent with the literature. Adusei (2015) revealed that effective and efficient mobilization of deposits increases the financial stability of banks. These findings are also supported by other studies (Shleifer and Vishny, 2010; Köhler, 2015; Ali et al., 2019). The possible explanation for our result is that Pakistani banks are not effectively mobilizing the customer's deposits, which caused instability or no effect on the financial stability of banks. The study also used the interaction of liquidity risk and credit risk and found no significant influence of it on financial stability.

Besides this, numerous studies find a negative relationship between bank size and their financial stability (Altaee et al., 2013; Laeven et al., 2014; Köhler, 2015; Ali and Puah, 2018). The present study also reports similar results, which are backed by agency theory that reports the negative effect of bank size on bank stability. Jensen and Meckling (1976) elaborated on the agency theory and argued that principles and agents of firms have discordant goals. Agents have the intention of increasing the size of the firm to enjoy the greater benefits. Hence, the increase in firm size, with the view of increasing power and influence, caused bad governance in large firms.

## Conclusion and Recommendations

The present study is an attempt to determine the factors explaining the financial stability of the banking sector in Pakistan. To end this, the present study used bank-specific factors, such as liquidity risk, credit risk, and funding risk, as determinants of the financial stability of the banks. Our study finds that liquidity risk and credit risk have a significant negative influence on the financial stability of commercial banks in Pakistan. Where funding risk has no significant influence on stability. Besides these, bank size has also a negative impact on bank stability, whereas ROA revealed a positive influence.

The findings of our study have several implications for policymakers and bank management in Pakistan. Banks management should establish policies that ensure secure loan granting and timely reimbursement from customers to minimize the credit risk. Besides this, the management should keenly observe the liquidity position. According to our study, liquidity risk reduces the financial stability of the banks. Furthermore, banks should also effectively mobilize their customer's deposits to attain financial stability.

Besides its several implications, the present study also has some limitations. The present study only used the 15 conventional banks. Future studies can use both conventional and Islamic banks and can compare the stability differences between these banks. Furthermore, the present study only used the bank specific-factors. Future studies can use both banks inside and outside factors as determinants of bank stability.

## Data Availability Statement

The original contributions presented in the study are included in the article/supplementary materials, further inquiries can be directed to the corresponding author/s.

## Author Contributions

ZC: supervison of the whole research study. MS: conceptualization, methodology, formal analysis, software, and writing the original draft. NA: supervision. MM: visualization, writing—review, and editing. SR and NA: proof reading and finalizing the draft. All authors contributed to the article and approved the submitted version.

## Funding

We are thankful to the National Natural Science Foundation of China for supporting this research under fund number 72163018.

## Conflict of Interest

The authors declare that the research was conducted in the absence of any commercial or financial relationships that could be construed as a potential conflict of interest.

## Publisher's Note

All claims expressed in this article are solely those of the authors and do not necessarily represent those of their affiliated organizations, or those of the publisher, the editors and the reviewers. Any product that may be evaluated in this article, or claim that may be made by its manufacturer, is not guaranteed or endorsed by the publisher.
